# A Lower Degree of PBMC L1 Methylation in Women with Lower Folate Status May Explain the MTHFR C677T Polymorphism Associated Higher Risk of CIN in the US Post Folic Acid Fortification Era

**DOI:** 10.1371/journal.pone.0110093

**Published:** 2014-10-10

**Authors:** Suguna Badiga, Gary L. Johanning, Maurizio Macaluso, Andres Azuero, Michelle M. Chambers, Nuzhat R. Siddiqui, Chandrika J. Piyathilake

**Affiliations:** 1 The Department of Nutrition Sciences, The University of Alabama at Birmingham, Birmingham, Alabama, United States of America; 2 Biosciences Division, Center for Cancer and Metabolism, SRI International, Menlo Park, California, United States of America; 3 The Department of Pediatrics, Division of Biostatistics and Epidemiology, The University of Cincinnati College of Medicine, Cincinnati, Ohio, United States of America; 4 The Department of Community Health Outcomes and System, The University of Alabama at Birmingham, Birmingham, Alabama, United States of America; University of Navarra, Spain

## Abstract

**Background:**

Studies in populations unexposed to folic acid (FA) fortification have demonstrated that MTHFR C677T polymorphism is associated with increased risk of higher grades of cervical intraepithelial neoplasia (CIN 2+). However, it is unknown whether exposure to higher folate as a result of the FA fortification program has altered the association between MTHFR C677T and risk of CIN, or the mechanisms involved with such alterations. The current study investigated the following in a FA fortified population: 1) The association between MTHFR C677T polymorphism and risk of CIN 2+; 2) The modifying effects of plasma folate concentrations on this association; and 3) The modifying effects of plasma folate on the association between the polymorphism and degree of methylation of long interspersed nucleotide elements (L1s), in peripheral blood mononuclear cell (PBMC) DNA, a documented biomarker of CIN risk.

**Methods:**

The study included 457 US women diagnosed with either CIN 2+ (cases) or ≤ CIN 1 (non-cases). Unconditional logistic regression models were used to test the associations after adjusting for relevant risk factors for CIN.

**Results:**

The 677CT/TT MTHFR genotypes were not associated with the risk of CIN 2+. Women with CT/TT genotype with lower folate, however, were more likely to be diagnosed with CIN 2+ compared to women with CT/TT genotype with higher folate (OR = 2.41, P = 0.030). Women with CT/TT genotype with lower folate were less likely to have a higher degree of PBMC L1 methylation compared to women with CT/TT genotype with higher folate (OR = 0.28, P = 0.017).

**Conclusions:**

This study provides the first evidence that the MTHFR 677CT/TT genotype-associated lower degree of PBMC L1 methylation increases the risk of CIN 2+ in women in the US post-FA fortification era. Thus, even in the post-FA fortification era, not all women have adequate folate status to overcome MTHFR 677CT/TT genotype-associated lower degree of L1 methylation.

## Introduction

Folate, a water soluble B vitamin naturally present in fruits and vegetables, acts as a critical methyl donor for several molecular pathways (DNA methylation, synthesis and repair) necessary for cellular replication and maintenance. Inadequate folate status has been associated with several adverse health conditions, with substantial evidence of a relationship between poor folate status and the risk of neural tube defects (NTDs) [1], [2]. To reduce the risk of NTDs and improve folate intake in women of child bearing age, mandatory fortification of enriched cereal flour with folic acid (FA) was initiated in 1998 by the United States (US) food and drug administration [3]. For almost a decade FA fortification has been accepted not only in the US but also in several countries such as Canada and Chile [4]. This has resulted in a population wide increase in the circulating concentrations of folate [3]. Even though the reviews of the safety and toxicity of folate that were published prior to initiation of FA fortification in the US concluded that FA is safe under most circumstances even at supra-physiologic amounts, concerns have been raised about possible adverse effects of current folate intakes on several diseases including cancer [5], [6], [7]. Despite these concerns, the debate continues about whether even more FA should be added to the food supply in the US. Currently, most of these debates and concerns are largely speculative with regard to human populations, as they are not based on sound scientific evidence. Therefore, there is an urgent need to address these issues by the scientific community since concerns raised are a critical barrier for this field to progress. Our results generated by observational studies conducted in the US during the post FA fortification era are suggestive of higher folate’s beneficial effects rather than adverse effects on the primary prevention of higher grades of cervical intraepithelial neoplasia (CIN 2+) [8], [9], [10], [11]. CIN 2+ are pre-cancerous lesions that lead to the development of invasive cervical cancers (ICC) found in millions of women worldwide due to infections with high-risk human papillomaviruses (HR-HPVs), their primary causative factor [12]. Further, our studies demonstrated that the mechanisms by which higher folate may exert protective effects against CIN 2+ are likely to be via folate’s positive influence on immune response or DNA methylation. These results suggest that folate may still be used as a chemopreventive agent for HPV related cancers or as an adjunct to enhance the efficacy of therapeutic HPV vaccines. However, as discussed below, the effect of genetic variants of genes in the folate metabolism pathway (FMP) on the risk of CIN or ICC in the presence of higher folate status is still unclear.

Only a limited number of studies have evaluated the association between genetic polymorphisms of genes involved in folate metabolism and risk of being diagnosed with cervical pre-cancerous lesions or ICC [13], [14], [15], [16], [17], [18], [19], [20], [21], [22]. All except one of the studies were conducted in populations unexposed to a mandatory FA fortification program. The focus of these studies has been primarily on the genetic polymorphism C677T in the methylene tetrahydrofolate reductase (MTHFR) gene that encodes a critical enzyme in the folate metabolic pathway which is required for the generation of methyl groups for DNA methylation reactions. The results of these studies have been inconsistent as they report either positive, negative or no association. Recent meta-analyses suggested that MTHFR C677T polymorphism may not be associated with the risk of CIN/CC in the overall studies but may be ethnicity specific [23], [24], [25], [26]. Since these studies were not comprehensive, such meta-analyses were unable to evaluate the modifying effects of folate on the association between the MTHFR C677T polymorphism and risk of CIN/ICC. However, there have been convincing reports on the interaction between folate intake or circulating concentrations of folate and MTHFR C677T polymorphism in relation to the risk of colorectal cancer or adenoma [27], [28]. These results suggested that folate status may alter the effects of MTHFR C677T polymorphism, resulting in the polymorphism being protective in the presence of high folate status or being a risk in the presence of inadequate folate status. Although some studies have evaluated the association between MTHFR C677T polymorphism and CIN in populations exposed to different levels of FA [13], [17], [21], a formal interaction between MTHFR C677T polymorphism and circulating concentrations of folate on the risk of CIN in the US post-FA fortification era has not been reported.

As MTHFR is known to have a critical role in DNA methylation, alterations in enzyme activity due to the MTHFR C677T polymorphism were demonstrated to alter DNA methylation in several studies. These studies have shown that the presence of MTHFR C677T polymorphism may result in global DNA hypomethylation; further, one study has observed that the MTHFR C677T polymorphism influences DNA methylation status through an interaction with folate status [29], [30]. However, these investigations were conducted in healthy individuals or individuals diagnosed with coronary atherosclerosis prior to US FA fortification. Genome wide DNA hypomethylation (entire genome, repetitive elements or transposons) may lead to genomic instability, and has been causally linked to various cancers [31], [32], [33]. We have demonstrated previously that global DNA methylation, as represented by the degree of methylation of the long interspersed nucleotide elements-1 (L1) in peripheral blood mononuclear cells (PBMCs), is a potential biomarker of CIN 2+, and is influenced by methyl donor micronutrients, specifically folate and vitamin B12 [34]. However, there have been no studies reported on the possible modifying effects of higher folate status due to exposure to FA fortification on the association between MTHFR polymorphic status and the degree of PBMC L1 methylation. The purpose of the current study was therefore to investigate the following in a population exposed to FA fortification: 1) The association between MTHFR C677T polymorphism and risk of CIN 2+; 2) The modifying effects of plasma folate concentrations on this association; and 3) The modifying effects of plasma folate on the association between MTHFR C677T polymorphism and the degree of PBMC L1 methylation, a documented biomarker of CIN risk.

## Methods

### Study Population

The study population consisted of 457 women enrolled in a prospective follow-up study funded by the National Cancer Institute (R01 CA105448, Prognostic Significance of DNA & Histone Methylation). All women enrolled in this study were diagnosed with abnormal Pap smear by the Health Department in Birmingham, Alabama and were referred to the colposcopy clinic at the University of Alabama at Birmingham (UAB), Birmingham, Alabama for further examination by colposcopically directed biopsy. Women were 19–50 years old, had no history of ICC or other cancer of the lower genital tract, no history of hysterectomy or destructive therapy of the cervix; were not pregnant, and were not using anti-folate medications such as methotrexate, sulfasalazine, or phenytoin. All women included in this study were positive for HR-HPVs (any one or more of 13 types of HR-HPVs, HPV 16, 18, 31, 33, 35, 39, 45, 51, 52, 56, 58, 59 and 68). Cases were women diagnosed with CIN 2+ (includes women with CIN 2, n = 77; CIN 3, n = 52; and carcinoma *in-situ* [CIS], n = 3) and non-cases were women diagnosed with CIN≤1 (includes normal cervical epithelium, n = 17; HPV cytopathic effect [HCE], n = 37; reactive nuclear enlargement [RNE], n = 56; CIN 1, n = 215). All participants have given written consent in the parent study (R01 CA105448, Prognostic Significance of DNA & Histone Methylation) to allowing the samples to be preserved and used for conducting future studies that investigate factors involved in modifying the risk of cervical cancer. The parent study consent form, which included the request for permission for sample use for future studies, was read to the study participant by the study coordinator to assure that the participants fully understood the content of the consent form. The participants were given time to read the consent form and clarify any questions they may have had prior to signing the form. All study participants of the parent study signed in favor of using samples for future studies. The parent and the current study were approved by the UAB Institutional Review Board (Protocol numbers F040126002 and X100205011 respectively).

### Data collection

Demographic information (age, race, level of education) and life style factors (smoking status, alcohol consumption, oral/hormone contraceptive use, parity, lifetime number of sexual partners) were obtained from the interviewer-administered validated questionnaires. A Center for Disease Control (CDC) physical activity questionnaire was used to obtain information on the level of physical activity. Weight and height were measured using standard procedures. BMI was calculated using the formula: weight in kilograms divided by height in meters squared.

### Specimen collection

Fasting blood samples collected in EDTA-containing blood collection tubes, which were kept at 4°C, were transported on ice to the laboratory within two hours of collection. In the laboratory, cervical suspensions were centrifuged and the resulting pellets were re-suspended in fresh PBS. Aliquots used for HPV genotyping were stored in PreservCyt solution at −20°C.

### DNA extraction

Blood samples were processed to isolate buffy coat and plasma. DNA from buffy coat was extracted by standard phenol-chloroform extraction. Extracted DNA was used for determining the genotype of the MTHFR C677T polymorphism and assessing the degree of PBMC L1 methylation.

### MTHFR C677T genotyping

The genotyping of the MTHFR C677T mutation, which involves the substitution of alanine with valine, was carried out using PCR-RFLP analysis. The exonic primer 5′-TGAAGGAGAAGGTGTCTGCGGGA-3′ and the intronic primer 5′-AGGACGGTGCGGTGAGAGTG-3′ were used to generate a 198 bp fragment through PCR amplification of the DNA [35]. Briefly, 100 ng of the DNA was amplified in 10 µl of the PCR mix containing 1X PCR buffer, 1.5 mM MgCl_2_, 3.75 U of Amplitaq Gold Polymerase (Applied Biosystems), 100 µmol/L of dNTP, and 0.5 µmol/L of each of the primers. PCR amplification was performed on a GeneAmp PCR system 9700 with the following conditions: initial denaturation at 94°C for 5 min, thirty five cycles of 94°C for 30 s, 62°C for 30 s and 72°C for 30 s, and a final extension at 72°C for 7 min. The PCR product of 198 bp was digested with 2.5 U of the restriction enzyme HinfI (New England Biolabs) at 37°C for 4 hrs to yield 175 bp and 23 bp products. The digested DNA fragments were separated on 3% agarose by gel electrophoresis and visualized using ethidium bromide and a Kodak Image Station 4000R, (Molecular Imaging System, Carestream Health, Inc). Genotypes of known samples were used as controls to ensure quality control of each run of the PCR assays. In addition, 5% of the samples were repeated and there was 100% concordance. We were successful in genotyping 455 samples. We were unable to genotype 2 samples, one case and one non-case.

### Determination of L1 methylation in PBMCs

Methylation analysis of the L1 promoter (GeneBank accession no. x58075) in PBMCs was determined using a pyrosequencing based methylation assay method, as described previously [11]. Briefly, bisulfite treatment of 1 µg of DNA extracted from buffy coat was completed using the EZ DNA methylation kit (Zymo Research, CA), and the converted DNA was eluted with 30 µl TE buffer. PCR reactions were carried out using forward (5′-TTTTTTGAGTTAGGTGTGGG-3′) and reverse-biotinylated (5′-biotin-TCTCACTAAAAAATACCAAACAA-3′) primers, as described [36]. The biotinylated PCR product, purified and made single-stranded to act as a template, was annealed to the pyrosequencing primer (5′- GGGTGGGAGTGAT-3′) (0.4 µM final concentration), and then was subjected to sequencing using an automatically generated nucleotide dispensation order for sequences to be analyzed corresponding to each reaction. The pyrograms were analyzed using allele quantification (AQ) mode to determine the proportion of C/T, and hence methylated and unmethylated cytosines at the targeted position(s). The reproducibility of the assay was satisfactory with a CV of 2.0–2.2%.

### Determination of plasma concentrations of micronutrients

Plasma concentrations of micronutrients were measured using protocols validated in the Nutrition Sciences laboratory at UAB. Briefly, plasma concentrations of folate were measured using the *Lactobacillus casei* microbiological assay, plasma concentrations of vitamin B12 using a competitive radio-binding assay (SimulTRAC-SNB, MP biomedicals), vitamin C by high performance liquid chromatography (Hitachi pump L-2100; EM Science HITACHI 655A-11 Liquid Chromatography; Waters 2487 Dual λ absorbance Detector) and total carotene using spectrophotometry (Jenway 7315 Spectrophotometer). All samples were stored at −80°C and assayed within 2–3 months.

### Statistical methods

Descriptive statistics were used to describe the study population based on the case status. Differences in the proportions and medians between cases and non-cases were tested using Pearson’s chi-square and the Kruskal-Wallis test respectively. Percentages were used to determine the distribution of the genotypes of the MTHFR C677T by self–reported race and case status. The assumptions of Hardy-Weinberg equilibrium (HWE) for the genetic polymorphism were tested in the controls using Pearson’s chi-square test. The difference in the median plasma concentrations of micronutrients by the genotypes was statistically tested using the Kruskal-Wallis test. Unconditional logistic regression models were used to test the association between genotypes of MTHFR C677T and risk of being diagnosed with CIN 2+ after adjusting for age (continuous), self-reported race (African American [AA] vs Caucasian American [CA]), level of education (less than high school education vs high school education or higher), BMI (≥25 vs <25 kg/m^2^), smoking status (ever smokers vs never smokers), alcohol consumption (ever vs never), physical activity (≥150 vs <150 min/week), lifetime number of sexual partners (≥5 vs <5), parity (≥1 live birth vs 0 live births), use of oral/hormone contraceptives (user vs non-user in the past two years) and median plasma concentrations of cancer protective micronutrients (folate, vitamin B12, vitamin C and total carotene). We assessed the association between MTHFR C677T polymorphism and risk of CIN 2+ using a co-dominant genetic model comparing those heterozygous (CT) and homozygous (TT) for minor alleles to those homozygous (CC) for the major allele, using dummy variables to obtain ORs and 95% CI, and Wald’s chi-square test to estimate the P-value. Based on previously reported functional effects of the genotypes of the polymorphism [37], the heterozygotes were combined with those homozygous for minor alleles to explore the dominant model (MTHFR C677T polymorphism CT or TT vs CC). The models were adjusted for relevant demographic and risk factors of ICC previously mentioned. We also tested the association between concentrations of plasma folate and risk of CIN 2+ after adjusting for relevant demographic and risk factors of ICC.

Regardless of the significance of the main effects of the MTHFR C677T polymorphism and the nutrient, gene–nutrient interactions were tested on the risk of CIN 2+. The change in the deviance between the main effects and interaction term, i.e., plasma folate X genotype of the polymorphism, was tested using the likelihood ratio test. The median of the plasma folate concentrations was based on the entire study population. The joint effects of the genotypes and plasma folate were tested on the additive scale using a common referent group. To estimate the joint effects of MTHFR C677T polymorphism and plasma folate the following combinations were created: CT or TT genotype and plasma folate ≥11.25 ng/mL, CT or TT genotype and plasma folate <11.25 ng/mL, CC genotype and plasma folate ≥11.25 ng/mL, and CC genotype and plasma folate <11.25 ng/mL. The referent category in this analysis was CT or TT genotype and plasma folate ≥11.25 ng/mL. The models testing the joint effects were adjusted for the relevant demographic and risk factors of ICC previously mentioned.

The associations between the MTHFR C677T polymorphism and PBMC L1 methylation were tested among non-cases. Exclusion of cases was necessary to avoid the possibility of reverse causation. Unconditional logistic regression models specified PBMC L1 methylation as the binary outcome of interest (≥ highest tertile vs < highest tertile) and MTHFR C677T polymorphism as the independent predictor of interest (homozygous for minor allele or heterozygous vs homozygous for major allele). Models were adjusted for covariates that are likely to influence the degree of PBMC L1 methylation - age, self-reported race, BMI, smoking status, alcohol consumption, physical activity, parity and use of oral/hormonal contraceptives. Similarly, we tested the association between concentrations of plasma folate and degree of PBMC L1 methylation after adjusting for relevant covariates.

The effect of gene-nutrient interactions on the degree of PBMC L1 methylation was tested. To estimate the joint effects of MTHFR C677T polymorphism and plasma folate on the degree of PBMC L1 methylation, the following combinations were created using the median plasma folate concentrations of the non-cases: CT or TT genotype and plasma folate ≥11.21 ng/mL, CT or TT genotype and plasma folate <11.21 ng/mL, CC genotype and plasma folate ≥11.21 ng/mL and CC genotype and plasma folate <11.21 ng/mL. The referent category in this analysis was CT or TT genotype and plasma folate ≥11.21 ng/mL. The models testing the joint effects were adjusted for covariates that are likely to influence the degree of PBMC L1 methylation.

We estimated the strength of each association by estimating the odds ratio (OR) and 95% confidence interval (CI) and its statistical significance using Wald’s chi-square test. All statistical analyses were two sided and were considered significant at P<0.05 and all analyses were carried out in JMP version 9.0 (SAS Institute, Cary, NC).

## Results

The description of the cases and non-cases based on demographics, lifestyle factors, and plasma micronutrient concentrations is shown in Table 1. The study population consisted of 132 cases and 325 non-cases. All women were of child bearing age (between 19–45 years) except for two women who were >45 years. As compared to non-cases, cases were significantly more likely to be smokers and have ≥1 live birth (P<0.001 and P = 0.005 respectively). None of the other variables such as age, self-reported race, level of education, BMI, physical activity, lifetime number of sexual partners, use of hormonal contraceptives and plasma concentrations of folate vitamin B12, vitamin C and total carotene were statistically different by case status.

**Table 1:** Demographic, lifestyle, nutritional factors and PBMC L1 methylation of the study population by case status.

Variables	Cases	Non-Cases	P-value
Total (N)	132 (29%)	325 (71%)	
Age (years) >23 years	68 (52%)	160 (49%)	0.658
Race (% African American)	78 (59%)	220 (68%)	0.082
BMI^a^ (% ≥25 kg/m^2^)	69 (53%)	202 (63%)	0.053
Smoking status (% ever smoker)	89 (67%)	162 (50%)	<0.001
Physical activity (% ≥150 min/week)	22 (17%)	65 (20%)	0.406
Parity (% ≥1 live births)	101 (77%)	204 (63%)	0.005
Life time number of sexual partners (% ≥5)	65 (50%)	138 (43%)	0.172
Oral/hormonal contraceptive use(% ever users)	105 (80%)	278 (87%)	0.084
**Median (interquartile range) concentration** **of micronutrients**			
Plasma folate (ng/mL)	11.28 (8.19–15.95)	11.21 (7.92–16.63)	0.906
Vitamin B12 (pg/mL)	385.82 (247.15–576.40)	406.72 (293.81–546.55)	0.413
Total carotenes (µg/mL)	85.11 (66.51–109.84)	87.13 (66.54–109.35)	0.665
Vitamin C (mg%)	15.09 (10.24–15.09)	14.44 (10.43–20.34)	0.735
**Median (interquartile range) of** **PBMC L1** b **methylation (%)**	60.3 (56.92–68.42)	62.25 (57.73–70.13)	0.054

a BMI - body mass index.

b PBMC L1 - peripheral blood mononuclear cells long interspersed nucleotide elements −1.

The distribution of the MTHFR genotypes by self–reported race is shown in Table 2. The distribution of genotypes in controls and cases were in HWE by self-reported race. The minor allele frequencies (MAF) in AA and CA were 0.08 and 0.32 respectively, consistent with a previous study [38].

**Table 2:** Distribution of MTHFR C677T polymorphism genotype by self-reported race and case status.

		MTHFR C677T polymorphism genotypes n (%)*		
Self-reported Race	Case Status	CC	CT	TT
Caucasian American	Cases	18(33%)	31(57%)	5(10%)
	Non-cases	53(50%)	43(41%)	9(9%)
African American	Cases	67(87%)	8(10%)	2(3%)
	Non-cases	184(84%)	35(16%)	0(0%)
All races	Cases	85(65%)	39(30%)	7(5%)
	Non-cases	237(73%)	78(24%)	9(3%)

*Allele frequencies of both cases and non-cases in Hardy-Weinberg equilibrium (HWE) for both races.

Both MTHFR C677T polymorphism and plasma folate concentrations independently were not significantly associated with CIN 2+ after adjusting for all known risk factors of ICC (OR = 1.24, 95% CI = 0.73–2.08, P = 0.420; OR = 0.89, 95% CI = 0.57–1.39, P = 0.609 respectively). However, we observed a statistically significant interaction between MTHFR polymorphism and plasma folate after adjusting for all relevant demographic and lifestyle risk factors of ICC (P interaction = 0.016) (Table 3). Women with CT or TT genotype and plasma folate concentrations <11.25 ng/mL were 2.4 fold more likely to be diagnosed with CIN 2+ compared to women with CT or TT genotype and plasma folate concentrations ≥11.25 ng/mL (OR = 2.41, 95% CI = 1.09–5.48, P = 0.030).

**Table 3:** Joint effect of MTHFR C677T and plasma folate concentration on the risk of CIN 2+.

MTHFR C677T and folate combination	Number of cases/non-cases	OR (95%CI)	P-value*
CT+TT & folate ≥11.25 ng/mL	18/44	1.00 (ref)	
CT+TT & folate <11.25 ng/mL	28/41	2.41 (1.10–5.48)	0.030
CC & folate ≥11.25 ng/mL	48/116	1.52 (0.73–3.25)	0.264
CC & folate <11.25 ng/mL	36/119	1.13 (0.51–2.54)	0.766
***P for interaction***		0.016	

*Adjusted for age, self-reported race, level of education, BMI, physical activity, smoking status, alcohol consumption, parity, lifetime number of sexual partners, use of oral/hormone contraceptives and circulating concentrations of vitamin C, total carotene and vitamin B12.

To understand the functional mechanism through which MTHFR C677T polymorphism is likely to increase the risk of CIN 2+, we evaluated the association between this polymorphism and the degree of PBMC L1 methylation, which was associated with CIN 2+ in a previously published study [11]. MTHFR C677T polymorphism and plasma folate concentrations independently were not significantly associated with PBMC L1 methylation (OR = 0.98, 95% CI = 0.53–1.80, P = 0.953; OR = 0.79, 95% CI = 0.47–1.33, P = 0.382 respectively). However, we observed a statistically significant interaction between MTHFR C677T polymorphism and plasma folate after adjusting for all relevant demographic and lifestyle risk factors of ICC (P = 0.030) (Table 4). Women with CT or TT genotype and lower plasma folate concentrations were ∼70% less likely to have higher PBMC L1 methylation compared to women with CT or TT genotype and higher plasma folate concentrations (OR = 0.28, 95% CI = 0.09–0.78, P = 0.017).

**Table 4:** Joint effect of MTHFR C677T and plasma folate concentration on PBMC L1 methylation among non-cases.

MTHFR C677T and folatecombination	Number of women with PBMC L1Methylation ≥68.5%/<68.5%	OR (95% CI)	P-value*
CT+TT & folate ≥11.21 ng/mL	22/21	1.00 (ref)	
CT+TT & folate <11.21 ng/mL	8/32	0.28 (0.09–0.78)	0.017
CC & folate ≥11.21 ng/mL	39/77	0.55 (0.24–1.25)	0.151
CC & folate <11.21 ng/mL	35/79	0.63 (0.27–1.47)	0.285
***P-value for interaction***	0.030		

*Adjusted for age, self-reported race, level of education, BMI, physical activity smoking status, parity, use of oral/hormone contraceptive and circulating concentrations of vitamin C, total carotene and vitamin B12.

## Discussion

The association between MTHFR C677T polymorphism, a key enzyme in the FMP, and risk of CIN or ICC has been studied in both the pre-FA fortification and in the early part of the post-FA fortification periods in the US [13], [17], [21]. A study conducted by Piyathilake et al. prior to fortification suggested an increased risk for cervical squamous intraepithelial lesions (SILs) in the presence of the TT or CT genotype of the MTHFR C677T polymorphism [13]. Another study by Goodman et al. prior to fortification showed a positive monotonic trend in the odds ratio of cervical SILs with the number of variant MTHFR T alleles [17]. In addition, they demonstrated that women with the TT or CT genotype of the MTHFR C677T polymorphism and with lower folate intake were at a higher risk for cervical SILs compared to women with the CC genotype and higher folate intake. However, in the Atypical Squamous Cells of Undetermined Significance- Low Grade Squamous Intraepithelial Lesion (ASCUS – LSIL) Triage Study (ALTS) conducted during the initial period of fortification, Henao et al. observed that women polymorphic for MTHFR C677T were less likely to have CIN 2 or 3 [21]. In the current study, we have examined the association between MTHFR C677T and the risk of CIN 2+ in a population exposed to FA fortification for 6–8 years in the US. Unlike studies prior to or during the early period (1998–2000) of fortification, we did not observe a significant association between MTHFR C677T polymorphism and risk of CIN 2+. A possible explanation for these inconsistencies may be attributed to differences in the level of exposure to folate of these populations which may have resulted in differential effects of this polymorphism on risk of precancerous lesions of the cervix. Similar inconsistencies have been reported for other cancers such as colorectal cancer [28]. Our observations suggested that genotype status alone may be insufficient to alter the risk for CIN 2+. Therefore, we investigated the joint effects of plasma folate concentrations and the same genetic polymorphism on the risk of CIN 2+. Our study results suggested that in the post-FA fortification era the polymorphism is protective only in the presence of higher plasma folate concentrations.

MTHFR is a rate limiting enzyme in the FMP that catalyzes the irreversible conversion of 5, 10 methylenetetrahydrfolate to 5-methyltetrahydrofolate required for remethylation of homocysteine [39]. A common missense mutation of C to T transition at the nucleotide base pair of MTHFR 677 resulting from the substitution of alanine to valine amino acid at position 222 makes this enzyme thermolabile [40]. The CT and TT genotypes of this polymorphism have 30% and 65% reduced activity compared to the CC genotype respectively [37]. The reduced activity of the MTHFR 677 TT and CT genotypes is due to the decreased efficiency of the polymorphic enzyme to bind the co-factor flavin adenine dinucleotide (FAD) effectively. One biologically plausible mechanism through which folate may modify the association between MTHFR C677T polymorphism and cancer risk is through effects on the efficiency of the polymorphism in binding FAD. A study evaluating the biochemical structure using an MTHFR polymorphic protein expressed in *E*.*coli* has shown that the polymorphic MTHFR possesses an exposed binding site for FAD that results in weak binding of the cofactor [41]. Ultimately this may lead to dissociation of the FAD and decreased activity of the enzyme. The presence of adequate 5-methyl tetrahydrofolate substrate brings about conformational changes in the MTHFR polymorphic enzyme that suppresses the disassociation of the FAD and prevents loss of activity [41]. The biological significance of the MTHFR C677T polymorphism is related to the reduced ability to provide 5-methyl tetrahydrofolate required for DNA methylation. Several studies have shown that individuals with TT genotype have diminished PBMC global DNA methylation compared to those with CC genotype [42], [43]. Further, we have previously demonstrated that lower PBMC L1 methylation, which is a surrogate marker of global DNA methylation status and a potential biomarker of susceptibility to cancer in other organs [44], [45], [46] and other obesity related disease conditions such as stroke, ischemic heart disease and cardiovascular diseases [47], [48], is also a potential biomarker for CIN 2+ [34]. These observations suggest that those with MTHFR C677T polymorphism and lower folate status are more likely to have lower PBMC L1 methylation, predisposing them to higher risk of CIN 2+. This is supportive of the modifying effects of higher folate on the association between MTHFR C677T polymorphism and risk of CIN 2+ observed in our study. Overall, our results suggest that higher folate may compensate for the reduced activity of the MTHFR C677T polymorphism and improve the degree of methylation in PBMCs, and in turn mount a protective immune response against HR-HPVs. The study of Hsiung et al. supports this idea by demonstrating that PBMC methylation has a positive influence on immune response against HPV-related head and neck cancer [45]. Therefore, our results suggest that the susceptibility to HR-HPV–related cervical carcinogenesis may also be an epigenetically modified process operating via a central mechanism.

Even though this study demonstrated that a higher folate status altered the association between MTHFR C677T and CIN 2+, a limitation of this study is that we did not identify additional polymorphisms of the MTHFR gene such as MTHFR A1298C that are or may be in linkage disequilibrium with MTHFR C677T polymorphism in our population, and therefore likely associated with disease risk. A few studies have demonstrated that MTHFR polymorphism A1298C with lower enzyme activity and in linkage disequilibrium with MTHFR C677T is likely to be associated with risk of CIN [49], [50]. Thus, evaluation of one polymorphism in the MTHFR gene limits our ability to conclusively associate the gene with the risk of CIN 2+. However, our observation that this commonly studied polymorphism in the presence of lower folate is associated with a lower degree of PBMC L1 methylation, a CIN 2+ associated biomarker, is an important finding of the study as it would allow other investigators to compare their study results and investigate the functional mechanism through which this polymorphism may operate to alter the risk of other cancers. It is likely that this association is due to the presence of other similar polymorphisms such as MTHFR A1298C. However, a recent study by Friso et al. that assessed the association between MTHFR A1298C and C677T gene polymorphisms and lymphocyte DNA methylation found that in individuals with lower circulating concentrations of folate, the negative association between MTHFR 1298AA wild type genotype and DNA methylation was driven by the concomitant presence of MTHFR C677T polymorphism [51]. Finally, the small sample size of our study was a limiting factor for analyzing the data by ethnicity. We are currently focused on addressing these limitations in our future studies by evaluating several SNPs of genes in the folate metabolic pathway using a larger sample size of a similar population.

Despite these limitations, this study has various strengths that add validity to our results. First, both cases and controls had a similar exposure to FA and the causative factor for CIN, as indicated by the fact that all women included in the study tested positive for HR-HPVs detected by a PCR-based method. Further, we have used histological diagnosis independently confirmed by two pathologists to categorize the population as cases and non-cases, ruling out the possibility of misclassification or inaccurate lesion diagnoses based on Pap test. Finally, our study population has been well characterized for known risk factors of ICC including plasma concentrations of “cancer protective micronutrients,” including vitamin B12, which has an important role in the FMP. This study is timely because of the significant concerns raised regarding the cancer modifying effects of population-wide exposure to higher folate in the US, and the highly controversial nature of arguments made about the positive or negative effects of the FA fortification program. The study provides the first evidence that the MTHFR CT/TT genotype-associated lower degree of PBMC L1 methylation increases the risk of CIN 2+ in women of child bearing age in the US post-FA fortification era. Even though the results of this study only apply to women of child-bearing age exposed to higher folate status, our findings are of significant public health importance for the following reasons. First, our study population consisted of women at risk for CIN, which makes up 14% of the US women of child-bearing age [52]. Therefore, results from the proposed study will apply to a significant proportion of US women and women with similar health issues world-wide. In addition, global DNA methylation in repetitive elements has also been associated with the risk of NTDs, CVDs and cancer [53], [49]. Therefore, our findings of an association between MTHFR C677T polymorphism and PBMC L1 methylation may be related to these health outcomes prevalent in these women. More importantly, the degree of PBMC L1 methylation may serve as a biomarker for identifying MTHFR C677T polymorphic women with low folate status, a high risk group of women for developing not only CIN 2+, but also other MTHFR polymorphism and low folate related health conditions. This study concludes that a lower degree of PBMC L1 methylation in MTHFR C677T polymorphic women with lower folate status may explain their higher risk of CIN 2+. Future studies are needed to evaluate the significance of lower PBMC L1 methylation in MTHFR C677T polymorphic women with lower folate status in relation to other health conditions common among women of child-bearing age.
